# Mapping the phylogeny and lineage history of geographically distinct BCG vaccine strains

**DOI:** 10.1099/mgen.0.001077

**Published:** 2023-08-01

**Authors:** Linzy Elton, Sandeep Kasaragod, Helen Donoghue, Hussain A. Safar, Priscilla Amankwah, Alimuddin Zumla, Adam A. Witney, Timothy D. McHugh

**Affiliations:** ^1^​ Centre for Clinical Microbiology, Division of Infection and Immunity, University College London, London, UK; ^2^​ Institute of Infection and Immunity, St George’s, University of London, London, UK; ^3^​ Genomics, Proteomics and Cellomics Sciences Research Unit (OMICSRU), Research Core Facility, Health Sciences Centre, Kuwait University, Kuwait City, Kuwait; ^4^​ National Institute for Health and Care Research Biomedical Research Centre, University College London, London, UK

**Keywords:** BCG, long read, Oxford Nanopore Technologies, strain, tuberculosis, vaccine, whole-genome sequencing

## Abstract

The bacillus Calmette–Guérin (BCG) vaccine has been in use for prevention of tuberculosis for over a century. It remains the only widely available tuberculosis vaccine and its protective efficacy has varied across geographical regions. Since it was developed, the BCG vaccine strain has been shared across different laboratories around the world, where use of differing culture methods has resulted in genetically distinct strains over time. Whilst differing BCG vaccine efficacy around the world is well documented, and the reasons for this may be multifactorial, it has been hypothesized that genetic differences in BCG vaccine strains contribute to this variation. Isolates from an historic archive of lyophilized BCG strains were regrown, DNA was extracted and then whole-genome sequenced using Oxford Nanopore Technologies. The resulting whole-genome data were plotted on a phylogenetic tree and analysed to identify the presence or absence of regions of difference (RDs) and single-nucleotide polymorphisms (SNPs) relating to virulence, growth and cell wall structure. Of 50 strains available, 36 were revived in culture and 39 were sequenced. Morphology differed between the strains distributed before and after 1934. There was phylogenetic association amongst certain geographically classified strains, most notably BCG-Russia, BCG-Japan and BCG-Danish. RD2, RD171 and RD713 deletions were associated with late strains (seeded after 1927). When mapped to BCG-Pasteur 1172, the SNPs in *sigK*, *plaA*, *mmaA_3_* and *eccC_5_* were associated with early strains. Whilst BCG-Russia, BCG-Japan and BCG-Danish showed strong geographical isolate clustering, the late strains, including BCG-Pasteur, showed more variation. A wide range of SNPs were seen within geographically classified strains, and as much intra-strain variation as between-strain variation was seen. The date of distribution from the original Pasteur laboratory (early pre-1927 or late post-1927) gave the strongest association with genetic differences in regions of difference and virulence-related SNPs, which agrees with the previous literature.

## Data Summary

All genome data for this study have been deposited in GenBank. Oxford Nanopore sequence reads were deposited under BioProject ID PRJEB61685. Accession numbers for previously published sequencing reads used to construct wider phylogenies are also in Table S3, available in the online version of this article. The authors confirm that all supporting data and protocols have been provided within the article or through supplementary data files.

**Impact Statement**

We describe a large collection of reference and experimental BCG vaccine strains both reference and experimental, many of which do not appear to have undergone whole-genome sequencing before. The availability of these novel sequence data for some strains will allow further analysis into potential vaccine targets in non-commercially available BCG strains. Genetic analysis provides a greater understanding of virulence factors and regions of difference and gives further insights into potential vaccine targets.

## Introduction

The *Mycobacterium bovis* bacillus Calmette–Guérin (BCG) vaccine has been in use for over 100 years and is currently the only widely available vaccine against tuberculosis (TB) [1]. BCG was developed as a commercial vaccine between 1924 and 1927 and distributed to at least 60 different laboratories around the world, continuing to be shipped to new laboratories into the 1940s [2]. This distribution led to the creation of multiple vaccine strains, as BCG is a live vaccine and required passaging every few weeks. At the time there was no standardization of culture and propagation techniques, and the strains were grown and maintained in varying conditions, leading to multiple genetically and phenotypically distinct daughter strains [3]. For the commercially available BCG vaccine strains there are detailed histories of their culture and storage conditions, including BCG-Japan 172 [4] and BCG-Russia [5], as well as historical reviews from Oettinger [2] and Osborn [6].

Differences in the efficacy of the BCG vaccines were soon identified. Years of research has shown this to be a complex picture and likely due to multiple factors, including host immunity [7, 8], concomitant diseases [9–11], geography and population age [12], vaccine dose and delivery [13, 14], growth conditions [15], differences in clinical trials methodology [16] and viability [17] of the vaccine strains used. With the advent of genetic techniques, attention turned to exploring whether the BCG strains’ genetic variation was a possible reason for the differences in efficacy [18]. Whilst each geographical strain has multiple unique single-nucleotide polymorphisms (SNPs), they can be broadly categorized as ‘early’ (pre-1927) and ‘late’ (post-1927). Early strains were exported from the original Pasteur culture before 1927 and include BCG-Russia [19], BCG-Sweden [20], BCG-Japan [21], BCG-Moreau [22] and BCG-Birkhaug [23]. These strains all contain the region of difference (RD) 2, which encodes secreted immunogenic proteins [18, 24].

Some potentially significant mutations that may affect vaccine efficacy appear to have occurred in the original French laboratory strain between 1927 and 1931 [25]. Strains exported after 1927 are termed late strains and include BCG-Frappier [22], BCG-Connaught [26], BCG-Mexico, BCG-Tice [27], BCG-China, BCG-Phipps, BCG-Prague, BCG-Glaxo, BCG-Merieux [28] and BCG-Danish [29], as well as a second BGC-Pasteur strain [30] (after a fire in the original laboratory, the mother strain was lost) [31]. Primarily, these late strains have lost RD2 and are generally thought to be less virulent than the early ones [7, 32–34]. Genes within the RD2 region play a role in cell wall synthesis, which may impact on virulence, although there does not appear to be a consensus as to how much of an effect this has, and whether late BCG strains are more attenuated and therefore show fewer side effects [24, 35, 36]. At the same time, a SNP occurred in *mmaA_3_*, a gene involved in methoxymycolic acid production, which appears to have affected BCG’s cell wall growth and function within macrophages, which may also alter vaccine efficacy [25].

Both *in vitro* and clinical studies have suggested that the genetic variations between BCG strains result in significant differences in gene expression, immunogenicity and virulence [37–40]. It is thought that with increasing passages over time, BCG strains may have become over-attenuated and less effective as a vaccine, as they are less able to prompt the immune system [41]. It has also been suggested that the early, more virulent, strains are also more likely to cause negative side effects, although there appears to be some disagreement in the literature, suggesting that other genetic losses may play a role [14, 34, 42, 43].

The concern that continuing differences in growth and storage methods were affecting the genetics of BCG strains prompted the World Health Organization’s (WHO’s) consultation on BCG vaccines in 1965 [44] and BCG vaccine standardization was brought in. Since then, vaccines have only been produced from lyophilized seed lots, and are to be no more than four generations from the original lot [45, 46]. BCG-Danish 1331, BCG-Japan 172–1 and BCG-Russia-1 are the WHO standardized reference strains [29].

**Fig. 1.**
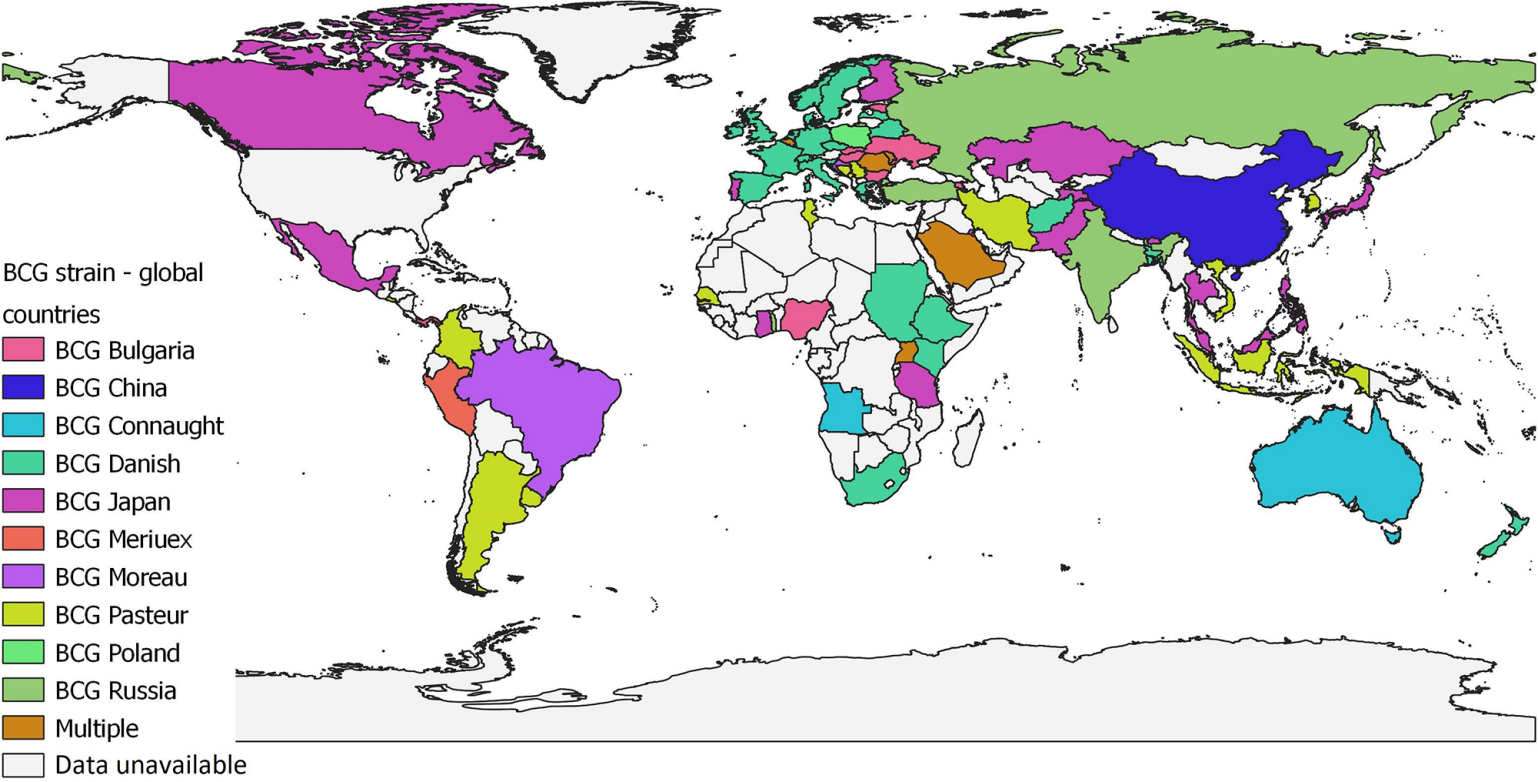
Map showing the global use of BCG vaccine strains, as of June 2021, based on data obtained from BCG World Atlas [50]. No data were available from countries coloured grey. Map produced using QGIS 3.8 Zanzibar.

Today, over 90 % of the BCG vaccines currently used worldwide are from six strains: BCG-Pasteur 1173 P2, BCG-Danish 1331, BCG-Glaxo 1077 (derived from the BCG-Danish strain), BCG-Japan 172–1, BCG-Russia BCG-I and BCG-Moreau RDJ strains, each of which is known to have different phenotypic characteristics [47, 48]. The majority of countries are supplied with three of these strains (BCG-Danish, BCG-Russia, BCG-Japan) on behalf of the Global Alliance for Vaccines and Immunization (GAVI), but a few countries, such as PR China, produce their own BCG vaccine [49]. Whilst most high-income countries (HICs) usually have a single strain licensed for use, many low- and middle-income countries (LMICs) may have several BCG vaccine strains circulating at any one time [7]. Fig. 1 shows the global usage of BCG strains, using data taken from BCG World Atlas [50, 51].

To provide long-term protection, vaccines should mimic natural infection immunologically without disease development. Even though BCG strains share >99 % of the *Mycobacterium tuberculosis* sequence, the multiple passages and different culturing methods resulted in an accumulation of genetic variations and the loss of important regions in BCG strains [52]. These genetic variations and deletions impacted on the strains both phenotypically and in their antigenicity, and several RD regions of tuberculosis have been identified as vaccine targets.

Whilst the more common BCG reference vaccine strains have been sequenced and genetic differences have been identified, some strains, especially experimental ones, have yet to be sequenced. As a result of the WHO’s BCG standardization meeting in the 1960s, the Middlesex Hospital Medical School, UK (now part of UCL) obtained a large collection of both commercially available reference and experimental BCG strains from around the world. This collection was saved and has been stored at the UCL Centre for Clinical Microbiology under the stewardship of Dr Helen Donoghue.

In this study we aimed to revive and whole-genome sequence the archival collection of BCG reference and experimental strains, which have not been passaged since the 1960s. Sequence data, supplemented with metadata obtained from the collection and the literature, were used to create a comprehensive picture of these strains, identify relatedness and identify any genetic variations that may relate to vaccine efficacy or safety.

## Methods

The collection of 50 geographical strains was recatalogued to ascertain the number of vials and any attached metadata, such as freeze dry date and production laboratory (see Table S1, available in the online version of this article). A random vial of each vaccine strain was chosen and, under sterile conditions, 500 µl sterile phosphate-buffered saline (PBS) (P4417-50TAB, Sigma Aldrich) was added. This was left to reconstitute for 5 min and then 200 µl was placed into an MGIT tube with BBL Middlebrook OADC Enrichment (Beckton Dickinson). One hundred microlitres was placed onto a Middlebrook 7H11 agar (no glycerol) slope (BM0781, E and O Laboratories Ltd). MGIT tubes were incubated in a BD BACTEC MGIT at 37 °C and agar slopes in a 37 °C static incubator. Growth in MGIT tubes was categorized morphologically.

If there was growth either in the MGIT tube or on the agar slope, DNA was extracted using the CTAB method as previously described [53]. If a strain failed to grow, DNA extraction was attempted directly from the vaccine vial (either from the original 200 µl if there was only one vial available, or from the full 500 µl if there was more than one vial). DNA concentration and molecular weight were checked using the Qubit dsDNA BR Assay kit (Thermo Fisher) and Genomic DNA ScreenTape and reagents on the TapeStation 4150 (Agilent Technologies, Inc.) to confirm the required quantity and quality for sequencing.

A DNA library was prepared using the Oxford Nanopore Technologies (ONT) Rapid PCR Barcoding kit (SQK-RPB004). The ONT kit protocol was followed [54], with the inclusion of a 0.6× AMPure bead (Beckman Coulter, Inc.) wash step prior to PCR amplification. Up to 12 barcoded strains were run together on a flow cell version R9.4.1 (ONT) using a MinION device for 48 h, using the default parameters on MinKNOW software (version 20.06.5). Basecalling was performed either by the MinKNOW software alongside sequencing or using the Guppy basecalling software (version 5.0.11), using the flip-flop fast algorithm. Sequence data were deposited under BioProject ID PRJEB61685.

Sequences were quality checked using FASTQC (v7.18.1) and MultiQC (v1.13) [55, 56] and then aligned to the BCG-Pasteur 1173 P2 (RefSeq: NC_008769) genome using Minimap2 (v2.24) [57] and sorted and indexed using samtools (v1.16.1) [58]. Site and variant calling were performed using bcftools (v1.16) mpileup, call and filter [59]. Sequence average depth of coverage data can be found in Table S2. Isolates with an average depth of 40× or more were processed for RD and SNP analysis. A score of 0 indicated that an SNP was not present, 1 indicated that an SNP was present but the quality was low (QUAL <30, DP <10, within 3 bp of another SNP or within 10 bp of an Indel), 2 indicated that an SNP had mixed variation (>10 reads not supporting the base call) and 3 indicated a high-quality SNP. Variant calls were annotated using SnpEff (version 5.0e) [60]. A review of the current literature on SNPs that confer greater virulence or affect growth or cell wall functionality in BCG enabled the identification of genes of interest across the whole genome, which may contribute to differences in vaccine efficacy across the geographical strains. RD region analysis was performed to identify the presence or loss of regions using RDscan [61]. Statistical analyses of data were performed using Prism version 9.4.1 (GraphPad).

A reference phylogenetic tree was built using simulated 300 bp paired end reads from 34 published BCG genomes using wgsim (v1.6) [62] (Table S3). As no ancestral BCG strain exists or was sequenced, the simulated reads were aligned against the reference BCG-Pasteur 1173 P2 (RefSeq: NC_008769) using BWA [63] and sites called with bcftools (v1.16) [59]. Sites were filtered using the following criteria: mapping quality (MQ) above 30; site quality score (QUAL) above 30; having at least four reads supporting to reference and alternative sites; minimum of 75 % of reads supporting site (DP4). Isolates sequenced in this study were combined and clustered with the reference sequences by calling variants from the ONT reads solely at the sites used to generate the reference tree, as previously described [64]. Phylogenetic reconstruction was performed using IQ-TREE 2 (v2.2.0-beta) [65] restricted to those models supported by raxml (the GTR+F model was selected) and branch support values were determined using 1000 bootstrap replicates [66]. The mapping reference (BCG-Pasteur 1173 P2) was specified as the outgroup.

## Results

### Growth and morphology of strains

An overview of the strains can be found in Table 1. Of the 50 BCG vaccine strains in the collection, 36 were grown successfully on at least one of the two growth media and DNA was extracted from a further 3 strains (directly from the vaccine vial). Of the 39 strains from which DNA was extracted, 25 were successfully sequenced to a depth of at least 40×.

**Table 1.** List of geographical strains represented in the collection, including the date they were seeded from the original Institut Pasteur strain, whether they are classified as early or late, and the MGIT tube morphology identified

Strain	Early/late strain	Date seeded from original Pasteur strain	Morphology (no./total grown in MGIT)	No. in collection	No. cultured	No. sequenced
Russia	Early	1924	Flake (2/2)	2	2	2
Japan	Early	1925	Flake (9/10)	12	10	10
Danish	Late	1931	Flake (7/9)	13	10	10
Prague	Late	1931 (seeded 1947 from Danish)	Flake (1/1)	2	1	1
Tice	Late	1934	n/a	2	0	1
Connaught	Late	1937 (seeded 1948 from Frappier)	n/a	2	0	1
Glaxo	Late	1931 (seeded 1954 from Danish)	Clump (2/2)	4	3	3
Pasteur	Late	1961	Clump (6/9)	10	8	9
Dakar	Late	1961*	Clump (1/1)	1	1	1
Dutch	Unknown	Unknown	n/a	1	0	1

*BCG-Dakar strain seeded from BCG-Pasteur 1961 strain, according to vial.

The morphology of each strain when grown in MGIT culture was recorded. Geographically classified early strains seeded from the original Institut Pasteur strain (BCG-Russia, BCG-Japan), BCG-Danish and BCG-Prague (seeded in 1947 from BCG-Danish) showed a flake morphology, whereas late strains (BCG-Glaxo, BCG-Pasteur and BCG-Dakar) showed a clump morphology, with the exception of BCG-Danish and Prague, as described above (see Fig. S3 for images).

### Strain relatedness

**Fig. 2.**
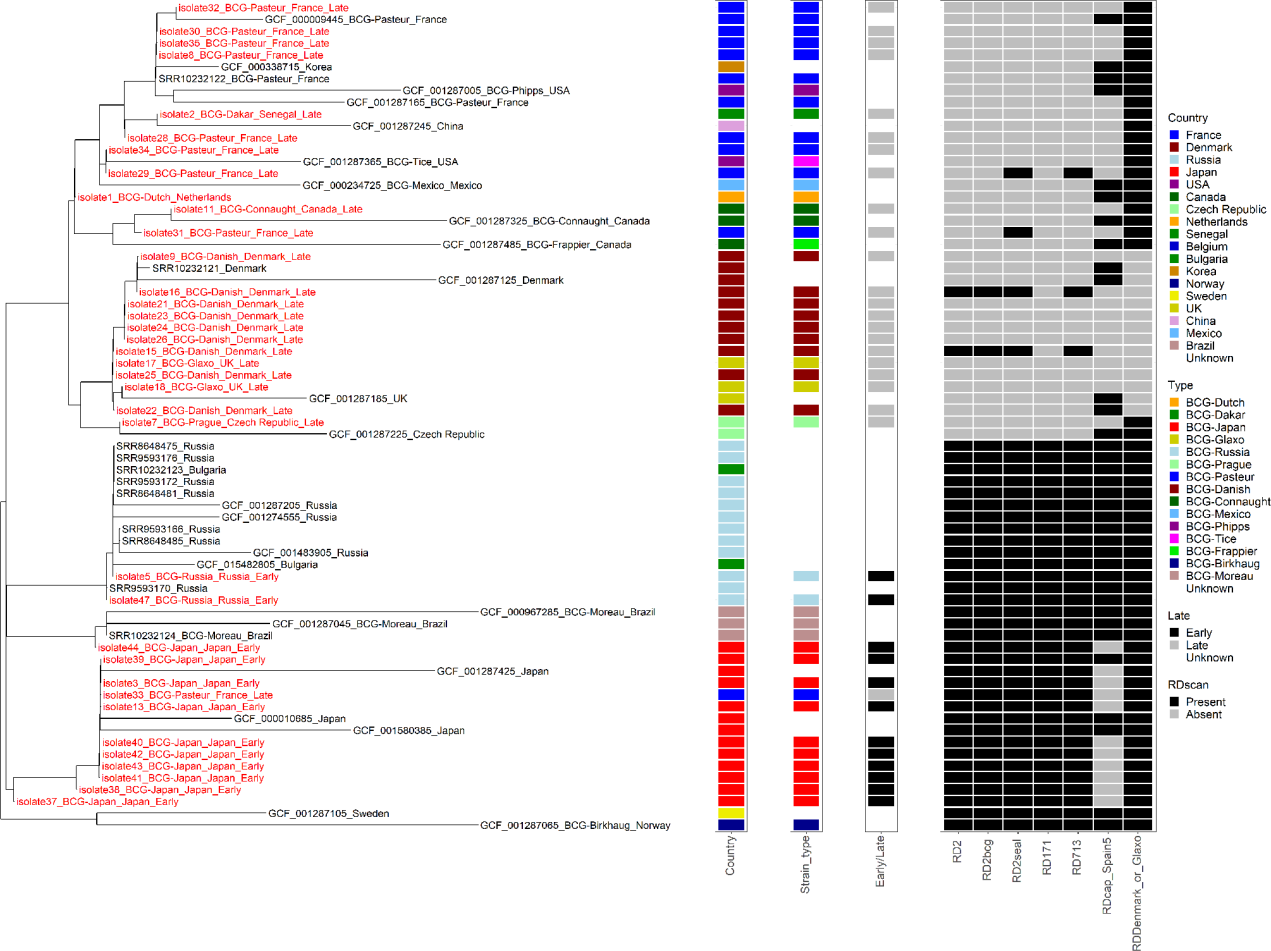
Phylogenetic tree of BCG vaccine strains sequenced in this study (red) and the reference strains (black). Those isolates sequenced within this study were combined and clustered with the reference sequences and are designated ‘isolate(number)_country_date_early/late’. The tree is rooted to the midpoint and branch lengths represent the number of substitutions per site. Reference genome names include the accession number and country. Colour coding for the country in which each isolate was cultured is shown in the left-hand column, showing the clustering of early strains; BCG-Russia, BCG-Moreau (reference strains only) and BCG-Japan. The late strains are located on a separate branch from the early strains and within that branch geographical isolates are separated into those distributed between 1931–1933, and those distributed after 1934. The middle column denotes whether isolates within this study were classed as early or late strains; early is shown in black and late in grey. The right-hand column shows the presence (denoted in black) or absence (in grey) of RD regions that appear to have some association with either early or late strains.

The phylogenetic tree displayed distinct separation of early and late vaccine strains with good correlation to geographical source (see Fig. 2). The early strains [BCG-Russia and BCG-Japan, and BCG-Moreau, BCG Sweden and BCG-Birkhaug (the latter three are only reference strains)] were located together on one side of the phylogenetic tree and showed strong geographical relatedness. The BCG-Russia cluster also included two reference BCG-Bulgaria strains. The reference strains (both distributed in 1926) for BCG-Sweden and BCG-Birkhaug cluster together within this branch.

The late strains were located on a separate branch from the early strains and within that branch geographical isolates were separated into those distributed between 1931–1933 and those distributed after 1934. Of those distributed between 1931–1933 (BCG-Danish, BCG-Prague and BCG-Glaxo), BCG-Prague formed its own cluster within this group, whilst BCG-Glaxo (UK) isolates were positioned within the BCG-Danish cluster. Strains distributed after 1934 [BCG-Tice, BCG-Phipps (both USA), BCG-Mexico, BCG-Pasteur and BCG-Dakar] all occurred together on a single branch of the tree, but the geographical clustering was less apparent, apart from BCG-Connaught and BCG-Frappier, both from Canada, which were also distributed after 1934 but form their own cluster within this branch. From this study, BCG-Dutch, designated as such within the laboratory strain collection, but for which there seems to be no reference in the literature, is located within the post-1934 cluster.

### Regions of difference

Of the 195 RDs examined, 84 were present in every strain sequenced in this study and 31 were absent across all strains sequenced. RDs that showed geographical strain-specific deletions are shown in Table 2. Despite the absence of RD2 previously reported as a defining feature of late strains, it was found in one of the seven BCG-Danish strains (an experimental rather than vaccine reference strain, classified as ‘strain 121’). All late strains were RD171− but RD2+, RD2bcg+, RD2seal+ and RD713+. One of the five BCG-Pasteur strains (designated 1173 reference strain, ‘batch A’) also contained RD2 and was RD171+, RD2+, RD2bcg+, RD2seal+ and RD713+. RD713 and RD171 were both present in all of the early geographical strains but generally not the late ones.

**Table 2.** Heatmap of the number and percentage of each geographical strain that had RD deletions. The majority of these RDs appear to have been lost alongside RD2 in 1927

	Early		Late			
	**Russia**	**Japan**	**Danish**	**Prague**	**Glaxo**	**Pasteur**
RD2	0 (0 %)	0 (0 %)	6 (86 %)	1 (100 %)	2 (100 %)	4 (80 %)
RD2bcg	0 (0 %)	0 (0 %)	6 (86 %)	1 (100 %)	2 (100 %)	4 (80 %)
RD2seal	0 (0 %)	0 (0 %)	6 (86 %)	1 (100 %)	2 (100 %)	4 (80 %)
RD171	0 (0 %)	0 (0 %)	7 (100 %)	1 (100 %)	2 (100 %)	4 (80 %)
RD713	0 (0 %)	0 (0 %)	6 (86 %)	1 (100 %)	2 (100 %)	4 (80 %)
Rdcap_Asia	1 (100 %)	7 (88 %)	5 (71 %)	1 (100 %)	2 (100 %)	5 (100 %)
Rdcap_Spain	1 (100 %)	8 (100 %)	7 (100 %)	1 (100 %)	2 (100 %)	5 (100 %)
RDGlaxo_Denmark	0 (0 %)	0 (0 %)	7 (100 %)	0 (0 %)	2 (100 %)	0 (0 %)
RD_Russia	0 (0 %)	8 (100 %)	7 (100 %)	1 (100 %)	2 (100 %)	5 (100 %)

Other notable RDs, but not related to early and late classification, include RDcap_spain5, which was present in BCG-Russia strains, but not in the other geographical strains, RDDenmark or Glaxo, which was present in all geographical strains except BCG-Danish, BCG-Connaught and BCG-Glaxo, and RD_Russia, which was present in all except BCG-Russia [61, 67].

**Fig. 3.**
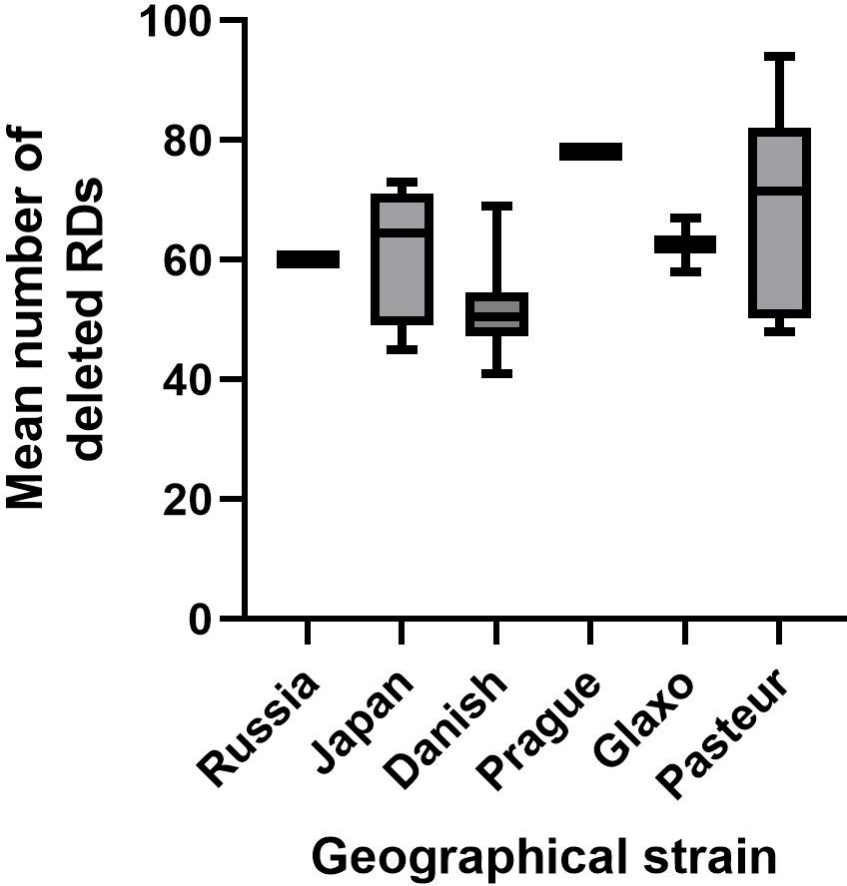
Box and whisker plot showing the mean number of deleted regions of difference for each geographical strain. There was a slight trend for increasing deletions over time (BCG-Russia earliest, BCG-Pasteur latest).

Whilst there was no significant difference in the mean number of deleted RDs between geographical strains when one-way analysis of variance (ANOVA) was applied, there was a trend to a slight increase relating to the time lapse before the geographical strain was taken from the original Institut Pasteur strain (see Fig. 3). The earliest strain, BCG-Russia, had 60 deleted RDs, whereas the latest, BCG-Pasteur, had a mean number of 69.

### SNP analysis

**Fig. 4.**
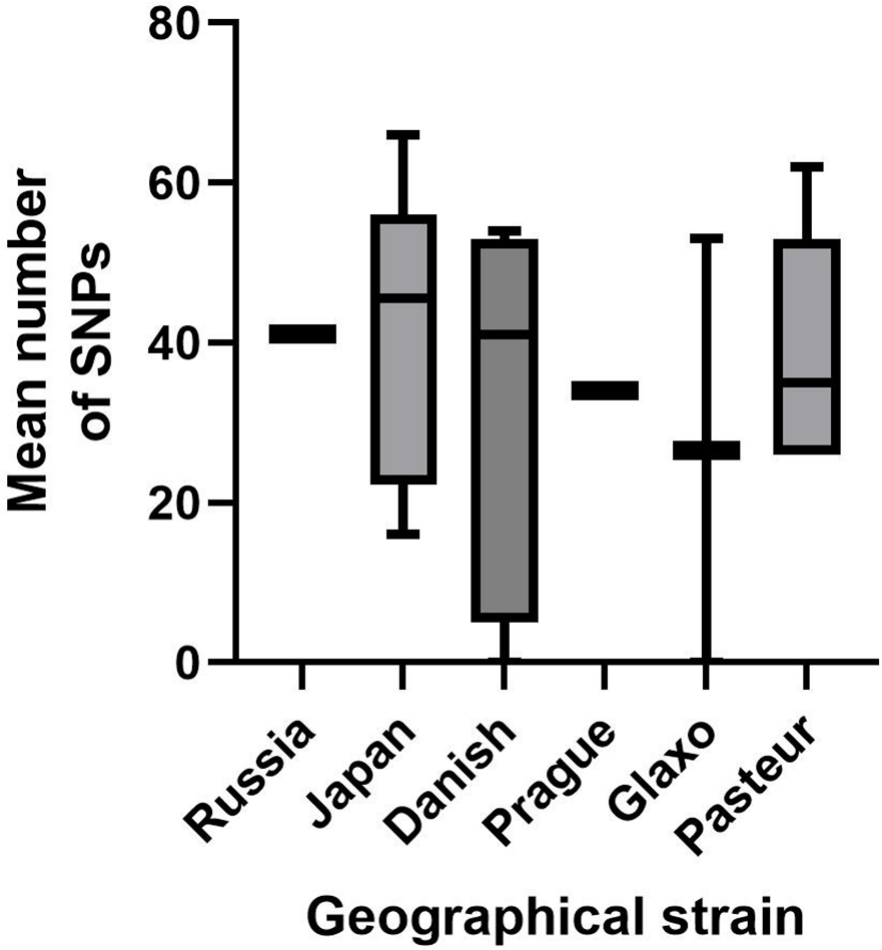
Box and whisker plot to show the mean number of high-quality SNPs (which scored 3), present across each geographical strain sequenced, when compared to the BCG-Pasteur 1173 P2 reference genome.

The mean number of total high-quality SNPs (as compared to the BCG-Pasteur 1173 P2 reference genome) was compared between the geographical strains and no significant difference was found when one-way ANOVA was applied (see Fig. 4). There was a large amount of variation in the mean number of SNPs in those strains in the archived laboratory collection with multiple isolates (BCG-Russia *n*=1, BCG-Japan *n*=8, BCG-Danish *n*=7, BCG-Prague *n*=1, BCG-Glaxo *n*=2 and BCG-Pasteur *n*=5).

BCG-Japan appeared to show the greatest number of SNPs, when compared to the BCG-Pasteur 1173 P2 reference genome, across genes related to virulence, growth or cell wall functionality, with at least one isolate having SNPs in multiple genes of interest (see Table 3). The early strains (BCG-Japan and BCG-Russia) exhibited SNPs in *sigK* (p.Ile1Met), *pcaA* (p.Thr154Pro), *mmaA3* (p.Asp98Gly) and either one or two in *eecC5* (p.Met647Ile and p.Val692Ala).

**Table 3.** Heatmap of the number and percentage of each geographical strain that exhibited SNPs in genes with virulence, growth or cell wall functionality, when compared to the BCG-Pasteur 1173 P2 reference genome

		Early			Late		
**Gene**	**SNP**	**Russia**	**Japan**	**Danish**	**Prague**	**Glaxo**	**Pasteur**
Mb0107	p.Glu189Gly	1 (100 %)	1 (13 %)	0 (0 %)	0 (0 %)	0 (0 %)	1 (20 %)
*tcrY*	p.Pro379Pro	0 (0 %)	2 (25 %)	2 (29 %)	0 (0 %)	1 (50 %)	3 (60 %)
*pepN*	p.Met18Ile	1 (100 %)	7 (88 %)	7 (100 %)	1 (100 %)	2 (100 %)	2 (40 %)
*sigK*	p.Ile1Met	1 (100 %)	8 (100 %)	0 (0 %)	0 (0 %)	0 (0 %)	1 (20 %)
*pcaA*	p.Thr154Pro	1 (100 %)	5 (63 %)	0 (0 %)	0 (0 %)	0 (0 %)	1 (20 %)
*mmaA3*	p.Asp98Gly	0 (0 %)	8 (100 %)	0 (0 %)	0 (0 %)	0 (0 %)	1 (20 %)
*eccC5*	p.Met647Ile	0 (0 %)	5 (63 %)	0 (0 %)	0 (0 %)	0 (0 %)	0 (0 %)
*eccC5*	p.Val692Ala	1 (100 %)	8 (100 %)	0 (0 %)	0 (0 %)	0 (0 %)	1 (20 %)
BCG_RS09420-BCG_RS09425	.	0 (0 %)	7 (88 %)	0 (0 %)	0 (0 %)	0 (0 %)	1 (20 %)
Mb2278c	p.Ala93Ala	0 (0 %)	7 (88 %)	0 (0 %)	0 (0 %)	0 (0 %)	1 (20 %)
Mb3159	p.Lys213Glu	1 (100 %)	7 (88 %)	0 (0 %)	0 (0 %)	0 (0 %)	1 (20 %)
*carD*	p.His13Arg	0 (0 %)	6 (75 %)	0 (0 %)	0 (0 %)	0 (0 %)	1 (20 %)
BCG_RS16215	p.Ser14Leu	0 (0 %)	0 (0 %)	6 (86 %)	0 (0 %)	2 (100 %)	0 (0 %)

## Discussion

The variation in the efficacy of BCG vaccination protocols has been widely discussed and there is evidence that vaccine strain variation is a contributing factor. Here we examined the genetic variation between strains in a historical archive, shedding light on the degree of genetic variation and specific differences that may impact on the protective immune response that is elicited.

The morphological variation between BCG strains that was observed is likely to be a product of both historical culture methods and variation in the expression of certain proteins. In 1983, Osborn *et al.* examined the culture of BCG strains (BCG-Danish 1331, BCG-Pasteur 1173, BCG-Japan 172 and BCG-Glaxo 1077) in different routine production techniques [6]. In their experiment, they found that different culture techniques yielded distinctive morphologies and that some vaccine preparations were heterogeneous and may have contained subpopulations. In this study, BCG strains were grown in standard conditions using MGIT tubes and two morphologies were noticed: flake and clump. Principally, early strains (BCG-Russia and BCG-Japan) and BCG-Prague and BCG-Danish showed flake morphology, while all other late strains (>1950 BCG-Glaxo, BCG-Pasteur and BCG-Dakar) grew as clumps. That there was a change in morphology occurring in strains distributed later suggests that morphology is likely linked to a genetic change that occurred around the 1940s in the original Institut Pasteur strain, although we did not observe any SNPs or RD deletions specific to these sets of geographical strains that may explain this change, suggesting a complex genetic picture.

Both the isolates sequenced within this study and the reference genomes also included in the phylogenetic tree show clustering that agrees with the chronological distribution from the original Pasteur laboratory and the early geographical strains (BCG-Russia and BCG-Japan) appear to show more defined clustering [68]. Russia was the first recorded daughter strain to be disseminated from the original Pasteur batch in 1924 and clustered with BCG-Bulgaria, which was seeded from BCG-Russia in the 1950s, after the original Pasteur strain they obtained caused unwanted side effects, so their association on the dendrogram is unsurprising [69, 70]. Similarly, there was an association between BCG-Danish and BCG-Glaxo on the dendrogram and this close genetic relationship is likely because BCG-Glaxo was seeded from BCG-Danish at the Statens Seruminstitut in 1954 [6].

Whilst the late strains distributed after 1934 were all located together in the same section of the dendrogram, there appeared to be less defined clustering of the geographical strains. BCG-Phipps, BCG-Tice, BCG-Korea and BCG-China (all reference genomes), and BCG-Dutch and BCG-Dakar (sequenced in this study) were located within the BCG-Pasteur strain cluster [2]. This lack of genetic variation in isolates distributed after 1934 compared to those distributed before may suggest the beginnings of awareness of vaccine efficacy differences and therefore of standardization in culture and vaccine production. That BCG-Dakar clusters with BCG-Pasteur helps to confirm the laboratory information provided, in that it was seeded from BCG-Pasteur at some point. BCG-Dutch, which was only designated ‘BCG-Dutch, vaccine A’ in the laboratory collection and for which we could find no information in the literature, also clustered with the BCG-Pasteur strains, suggesting that it could have been an experimental vaccine strain seeded from BCG-Pasteur, but never put into circulation as a commercial vaccine. The BCG-Pasteur strain designated ‘batch A’ in the archived collection appeared to be a potential anomaly, as it was located within the BCG-Japan cluster on the tree and showed differences in RDs and SNPs compared to other BCG-Pasteur isolates. This suggests that it is potentially a BCG-Japan strain that could have been mislabelled during the assembly of the archived laboratory collection.

In order to provide long-term protection, vaccines should mimic the natural infection immunologically without disease development. Even though BCG strains share >99 % of the *M. tuberculosis* genome, the multiple passages and different culturing methods resulted in accumulation of genetic variations and loss of important regions in BCG strains [52]. These genetic variations and deletions impacted on the strains both phenotypically and in their antigenicity. In this study, 84 RDs were present across all of the geographical strains sequenced in this study, and thus may be of use as potential vaccine candidates, with a number identified as vaccine targets [71, 72]. The presence or absence of five RDs and four SNPs related to virulence, growth or cell wall function appeared to separate early and late BCG strains. RD171 and RD713 were present in all early strains and absent in most late strains. Previous analyses by other groups suggest that RD713 overlaps with RD2 and RD2seal overlaps with RD713, which is also present in early and absent in late strains [61, 73–75]. However, in this study some late strains possessed these RDs that are typically associated with early strains and may reflect an unclear provenance for these strains. In *M. tuberculosis *, RD171 contains Rv1982A, which encodes the antitoxin VapB36, which may contribute to greater virulence and therefore immune response [76, 77].

SNPs were more likely to be seen in early strains, as the reference to which they were all mapped was BCG-Pasteur 1173 P2 [78], a late strain, due to the fact no ancestral BCG strain exists or has been sequenced. There was variation in the range of the numbers of SNPs found for each geographical strain, but this should be viewed with caution, asa different number of isolates was sequenced per geographical strain, in addition to the varying read depths obtained.

Early strains exhibited SNPs that appear to confer greater virulence, which agrees with the previous literature [41–43]. This includes *pcaA*, which encodes a mycolic acid cyclopropane synthase and plays a role in methyltransferase activity [79], and *mmaA_3_,* with a change in cell wall structure in late BCG strains [80, 81], both of which have been extensively reviewed. There was also a genetic difference in *sigK*, a positive regulator of the antigenic proteins MPT70 and MPT83 [78, 82]. Mutations in *sigK* in *M. tuberculosis* show variable production of these proteins and MPT70, an antigen unique to the *Mycobacteria*, is only produced in large quantities by BCG-Russia, BCG-Japan and BCG-Moreau, and produced in lower quantities in later strains and in *M. tuberculosis* [83]. In *M. tuberculosis,* MPT83, a surface lipoprotein, was shown to be one of the strongest Th1 cell antigens [84]. Possession of the early version of the *sigK* gene may therefore play a role in the greater immune response believed to be elicited by the early BCG strains.

Two variations were identified in the early strains in *eccC_5_*, a lesser described gene in BCG. *eccC*_5_ encodes a protein in the ESX-5 membrane complex secretion system [85, 86]. The ESX-5 secretion system is fundamental to *M. tuberculosis *–host cell interactions, related to its important role in PPE protein secretion, cell wall stability and virulence [85]. The ESX-5 secretion system only appears to have been described in detail in BCG in the BGC-Tice strain, which has a duplicated ESX-5 region [87]. Deletion of the ESX-5 type VII secretion system from *M. tuberculosis* is being tested as a vaccine candidate [88].

In this study we opted to use long-read ONT methodology and when these sequence data were compared to sequences simulated from reference genomes generated by Illumina sequencing within the phylogenetic tree, they interspersed as expected, suggesting that the data outputted from both platforms were comparable. However, the higher error rates in the ONT data may make the placement on the tree less reliable. Whilst short-read sequencing is well established and accounts for the vast majority of genomes uploaded to online databases, there are a number of advantages to long-read technologies when it comes to building detailed genomes of traditionally hard-to-sequence organisms. An especially important use of long-read sequencing is for building genomes with long runs of repeating sequences, such as in *M. tuberculosis* and BCG, and for this study it was especially advantageous in identifying the RD regions. Longer reads are also advantageous for *de novo* assembly. Additionally, the set-up cost of ONT platforms is much lower than, for instance, an Illumina MiSeq, which, combined with the portability of the devices, makes the MinION platform more accessible in resource-constrained settings.

## Conclusion

Whilst BCG-Russia, BCG-Japan and BCG-Danish showed strong geographical isolate clustering, the late strains distributed after 1934, including BCG-Pasteur, showed more widespread distribution. A wide range of SNPs were seen within geographically classified strains, and as much intra-strain variation as between-strain variation was seen. The greatest number of virulence-related SNPs and regions of difference were identified in those strains classed as early (which had a date of distribution before 1927 from the original Pasteur laboratory). This suggests, in agreement with the previous literature, that early strains may be more virulent and therefore likely to elicit a greater immune response in the host.

## Supplementary Data

Supplementary material 1
