# Supplementary material for: Mapping the phylogeny and lineage history of geographically distinct BCG vaccine strains
Source: Microb Genom. 2023 Aug 1;9(8):mgen001077. doi: 10.1099/mgen.0.001077 (PMC10483423; doi:10.1099/mgen.0.001077)
Supplement: Supplementary material 1 [file mgen-9-1077-s001.pdf]

Strain	Sub-strain	Reference or experimental strain	Freeze dry date	Early/ late	Morph. In MGIT	Growth on 7H11 slope	Growth in MGIT	CTAB extraction performed	Isolate name
BCG-Dutch	Dutch	Reference	Unknown	Early	n/a	No	No	Yes	Isolate1
BCG-Russia	Moscow strain	Reference	Unknown	Early	Flake	Yes	Yes	Yes	Isolate5
	Soviet strain	Unknown	Unknown	Early	Flake	Yes	Yes	Yes	Isolate47
BCG-Japan	Tokyo	Reference	Unknown	Early	Flake	Yes	Yes	Yes	Isolate3
	Reference vaccine, Japanese	Reference	Unknown	Early	Flake	Yes	Yes	Yes	Isolate13
	WHO	Reference	Unknown	Early	Flake	Yes	Yes	Yes	Isolate37
	172, batch C9	Reference	26/10/1976	Early	Clump	Yes	Yes	Yes	Isolate38
	172, batch A	Reference	03/10/1960	Early	Flake	Yes	Yes	Yes	Isolate39
	172, batch B	Reference	Unknown	Early	Flake	Yes	Yes	Yes	Isolate40
	172, batch C	Reference	Unknown	Early	Flake	Yes	Yes	Yes	Isolate41
	172, batch D	Reference	Unknown	Early	Flake	Yes	Yes	Yes	Isolate42
	172, batch E	Reference	Unknown	Early	Flake	Yes	Yes	Yes	Isolate43
	WHO, batch C	Unknown	Unknown	Early	Flake	Yes	Yes	Yes	Isolate44
	WHO, batch D	Unknown	Unknown	Early	n/a	No	No	No	n/a
	WHO, batch F	Unknown	Unknown	Early	n/a	No	No	No	n/a
BCG-Danish	121	Experimental	Unknown	Late	Flake	Yes	Yes	Yes	Isolate9
	122	Experimental	Unknown	Late	n/a	No	No	No	n/a
	121 (Tom Osborn)	Experimental	Unknown	Late	Clump	Yes	Yes	Yes	Isolate15
	Copenhagen strain, batch F10/A, made in England	Experimental	27/02/1962	Late	Flake	Yes	Yes	Yes	Isolate16
	Former Danish strain	Experimental	16/02/1967	Late	n/a	Yes	No	Yes	Isolate20
	1331, sub-lot A	Reference	29/09/1960	Late	Flake	Yes	Yes	Yes	Isolate21
	1331, sub-lot B	Reference	29/09/1960	Late	Flake	Yes	Yes	Yes	Isolate22
	1331, sub-lot C	Reference	29/09/1960	Late	Flake	Yes	Yes	Yes	Isolate23
	1331, sub-lot D	Reference	29/09/1960	Late	Clump	Yes	Yes	Yes	Isolate24
	1331, sub-lot E	Reference	29/09/1960	Late	Flake	Yes	Yes	Yes	Isolate25

	WHO	Reference	Unknown	Late	Flake	Yes	Yes	Yes	Isolate26
	batch 32	Unknown	Unknown	Late	n/a	No	No	No	n/a
	SSI, Copenhagen	Unknown	Unknown	Late	n/a	No	No	No	n/a
BCG-Prague	Czechoslovakian strain	Reference	Unknown	Late	Flake	Yes	Yes	Yes	Isolate7
	Czechoslovakian strain, (from BCG-Japan)	Unknown	Unknown	Late	n/a	No	No	No	n/a
BCG-Tice	USA, Chicago strain, IL105(s)50	Unknown	Unknown	Late	n/a	No	No	Yes	Isolate48
	USA, Chicago strain, 7498	Unknown	Unknown	Late	n/a	No	No	No	n/a
BCG-Connaught	Connaught strain, Toronto	Reference	Unknown	Late	n/a	No	No	Yes	Isolate11
	Diluent from Connaught	Unknown	Unknown	Late	n/a	No	No	No	n/a
BCG-Glaxo	French (Institut Merieux Lyon) strain, derived from Glaxo	Unknown	Unknown	Late	n/a	Yes	No	Yes	Isolate4
	Glaxo, Greenford	Reference	Unknown	Late	n/a	No	No	No	Isolate6
	1331/121, batch no. C8	Unknown	20/10/1976	Late	Clump	Yes	Yes	Yes	Isolate17
	1077/597, batch no. 295	Unknown	Unknown	Late	Clump	Yes	Yes	Yes	n/a
BCG-Pasteur	French (Institut Pasteur Paris)	Unknown	Unknown	Late	Clump	Yes	Yes	Yes	Isolate8
	WHO	Reference	Unknown	Late	Flake	Yes	Yes	Yes	Isolate28
	1173 P58	Reference	04/02/1968	Late	Clump	Yes	Yes	Yes	Isolate29
	1173, batch C10, P2 Pasteur strain	Reference	27/10/1976	Late	Clump	Yes	Yes	Yes	Isolate30
	1138	Experimental	Unknown	Late	n/a	No	No	Yes	Isolate31
	Batch 203	Unknown	Unknown	Late	Clump	Yes	Yes	Yes	Isolate32
	Pasteur, batch A	Unknown	Unknown	Late	Flake	Yes	Yes	Yes	Isolate33
	Pasteur, batch B	Unknown	Unknown	Late	Clump	Yes	No	Yes	Isolate34
	Pasteur, batch E	Unknown	Unknown	Late	Clump	Yes	Yes	Yes	Isolate35
	Pasteur, batch F	Unknown	Unknown	Late	n/a	No	No	No	n/a
BCG-Brussels	Institut Pasteur Brabent, Brussels	Unknown	Unknown	?	n/a	No	No	No	n/a
BCG-Dakar	Dakar strain	Unknown	Unknown	Late	Clump	Yes	Yes	Yes	Isolate2

Table S1. List of strains in the historic, lyophilised laboratory collection. Total number of strains in the collection = 50, total number successfully cultured = 35, total number successfully extracted = 39, total successfully sequenced = 38.

Isolate name	Strain	Sub-strain	Average depth
Isolate1	Dutch	Dutch	3.9
Isolate 2	Dakar	Dakar strain	14.2
Isolate 3	Japan	Tokyo	60.8
Isolate 4	Glaxo	French (Institut Merieux Lyon) strain, derived from Glaxo	0.08
Isolate 5	Russian	Russian strain, Moscow	16.5
Isolate 7	Prague	Czeckoslovakian strain (Prague)	1550.3
Isolate 8	Pasteur	French (Institut Pasteur Paris)	341.3
Isolate 9	Danish	121	769.2
Isolate 11	Connaught	Connaught strain, Toronto	26.8
Isolate 13	Japan	Reference vaccine, Japanese	1482.8
Isolate 15	Danish	121 (Tom Osborn)	49.1
Isolate 16	Danish	Copenhagen strain, batch F10/A, made in England	18.04
Isolate 17	Glaxo	1331/121, batch no. C8	50.4
Isolate 18	Glaxo	1077/597, batch no. 295	131.7
Isolate 20	Danish	Former Danish strain	1.9
Isolate 21	Danish	1331, sub-lot A	335.7
Isolate 22	Danish	1331, sub-lot B	23.9
Isolate 23	Danish	1331, sub-lot C	226.5
Isolate 24	Danish	1331, sub-lot D	345.4
Isolate 25	Danish	1331, sub-lot E	51.2
Isolate 26	Danish	WHO	220.9
Isolate 28	Pasteur	WHO	33.8
Isolate 29	Pasteur	1173 P58	4.3
Isolate 30	Pasteur	1173, batch C10, P2 Pasteur strain	268.2
Isolate 31	Pasteur	1138	7.5
Isolate 32	Pasteur	batch 203	29.3

Isolate 33	Pasteur	batch A	260.6
Isolate 34	Pasteur	batch B	287.9
Isolate 35	Pasteur	batch E	82.6
Isolate 37	Japan	WHO	7.7
Isolate 38	Japan	172, batch C9	69.1
Isolate 39	Japan	172, batch A	17.2
Isolate 40	Japan	172, batch B	242.8
Isolate 41	Japan	172, batch C	64.3
Isolate 42	Japan	172, batch D	83.07
Isolate 43	Japan	172, batch E	358.6
Isolate 44	Japan	WHO batch C	77.7
Isolate 47	Russian	Soviet strain	125.6
Isolate 48	Tice	USA, Chicago strain, IL105(s)50	0.06

Table S2. List of isolates extracted and sequenced in this study and the average sequencing depth. Project number PRJEB60755.

RefSeq_assembly_id	Genome_accession	Description	Included in tree

			generation (Y/N)
GCF_000009445	NC_008769.1	*Mycobacterium tuberculosis* variant *bovis* BCG str. Pasteur 1173P2, complete sequence	Y
GCF_000010685	NC_012207.1	*Mycobacterium tuberculosis* variant *bovis* BCG str. Tokyo 172, complete sequence	Y
GCF_000234725	NC_016804.1	*Mycobacterium tuberculosis* variant *bovis* BCG str. Mexico, complete sequence	Y
GCF_000338715	NC_020245.2	*Mycobacterium tuberculosis* variant *bovis* BCG str. Korea 1168P, complete sequence	Y
GCF_000967285	NZ_AM412059.1	*Mycobacterium tuberculosis* variant *bovis* BCG str. Moreau RDJ isolate SL.1 FAP RJ Passage B8S2 vaccine culture chromosome, complete genome	Y
GCF_001043255	NZ_CP008744.1	*Mycobacterium tuberculosis* variant *bovis* BCG strain 32.1 chromosome, complete genome	Y
GCF_001274555	NZ_CP009243.1	*Mycobacterium tuberculosis* variant *bovis* BCG strain Russia 368 chromosome, complete genome	Y
GCF_001287005	NZ_CUWN01000001.1	*Mycobacterium tuberculosis* variant *bovis* BCG strain Phipps, whole genome shotgun sequence	Y
GCF_001287045	NZ_CUWK01000001.1	*Mycobacterium tuberculosis* variant *bovis* BCG strain Moreau, whole genome shotgun sequence	Y
GCF_001287065	NZ_CUWE01000001.1	*Mycobacterium tuberculosis* variant *bovis* BCG strain Birkhaug, whole genome shotgun sequence	Y
GCF_001287105	NZ_CUWP01000001.1	*Mycobacterium tuberculosis* variant *bovis* BCG strain Sweden, whole genome shotgun sequence	Y
GCF_001287125	NZ_CUWH01000001.1	*Mycobacterium tuberculosis* variant *bovis* BCG strain Danish, whole genome shotgun sequence	Y
GCF_001287165	NZ_CUWL01000001.1	*Mycobacterium tuberculosis* variant *bovis* BCG strain Pasteur, whole genome shotgun sequence	Y
GCF_001287185	NZ_CUWJ01000001.1	*Mycobacterium tuberculosis* variant *bovis* BCG strain Glaxo, whole genome shotgun sequence	Y
GCF_001287205	NZ_CUWO01000001.1	*Mycobacterium tuberculosis* variant *bovis* BCG strain Russia, whole genome shotgun sequence	Y
GCF_001287225	NZ_CUWM01000001.1	*Mycobacterium tuberculosis* variant *bovis* BCG strain Prague, whole genome shotgun sequence	Y
GCF_001287245	NZ_CUWG01000001.1	*Mycobacterium tuberculosis* variant *bovis* BCG strain China, whole genome shotgun sequence	Y
GCF_001287325	NZ_CUWF01000001.1	*Mycobacterium tuberculosis* variant *bovis* BCG strain Connaught, whole genome shotgun sequence	Y
GCF_001287365	NZ_CUWQ01000001.1	*Mycobacterium tuberculosis* variant *bovis* BCG strain Tice, whole genome shotgun sequence	Y
GCF_001287425	NZ_CUWR01000001.1	*Mycobacterium tuberculosis* variant *bovis* BCG strain Japan, whole genome shotgun sequence	Y
GCF_001287485	NZ_CUWI01000001.1	*Mycobacterium tuberculosis* variant *bovis* BCG strain Frappier, whole genome shotgun sequence	Y
GCF_001293105	NZ_CP011455.1	*Mycobacterium bovis* strain BCG.1 genome	N
GCF_001483905	NZ_CP013741.1	*Mycobacterium tuberculosis* variant *bovis* strain BCG.1 (Russia) chromosome, complete genome	Y
GCF_001580385	NZ_CP014566.1	*Mycobacterium tuberculosis* variant *bovis* BCG str. Tokyo 172 chromosome, complete genome	Y
GCF_003703995	NZ_CP033310.1	*Mycobacterium tuberculosis* variant *bovis* BCG strain BCG-S48 chromosome	N
GCF_003704015	NZ_CP033311.1	*Mycobacterium tuberculosis* variant *bovis* BCG strain BCG_S49 chromosome	Y
GCF_015482805	NZ_CP064405.1	*Mycobacterium tuberculosis* variant *bovis* BCG strain BCG SL 222 Sofia chromosome, complete genome	Y

Table S3. BCG reference genomes used in this project, which were obtained from the NCBI database, and their NCBI accession numbers.

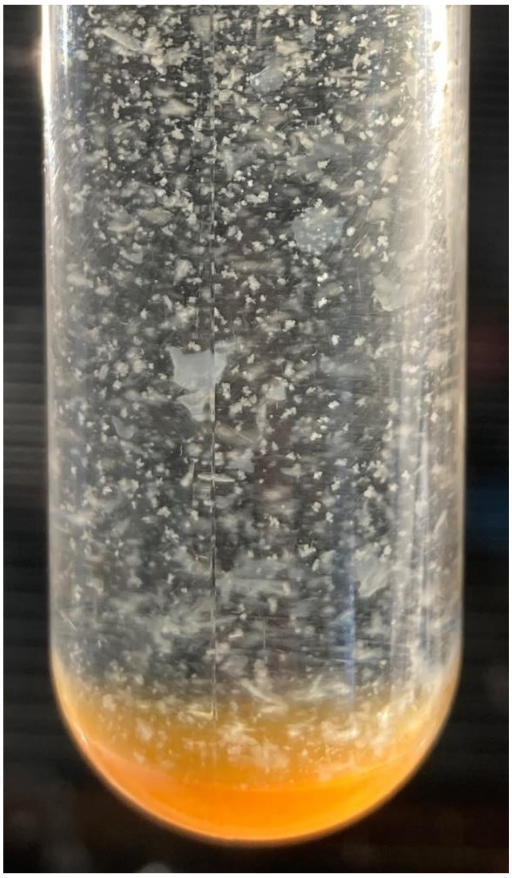

BCG-Russia (flake)

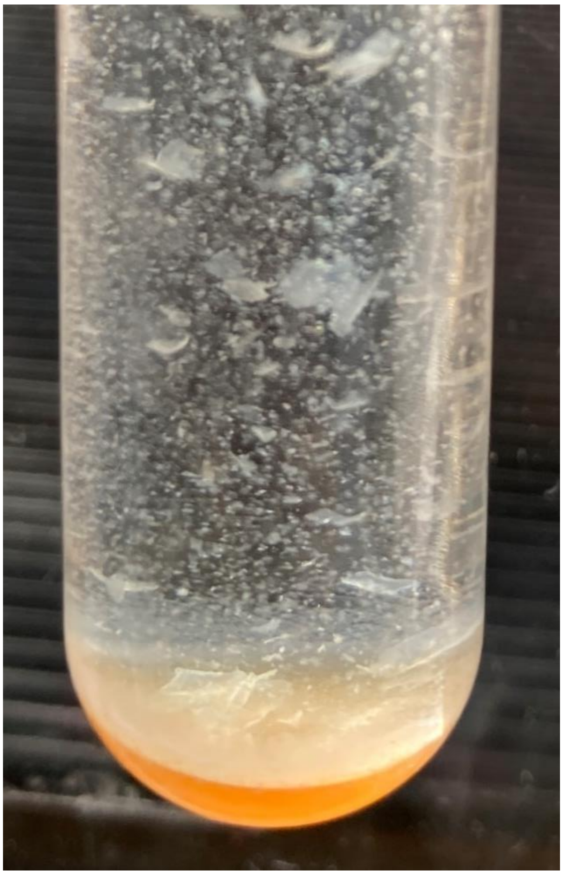

BCG-Japan (flake)

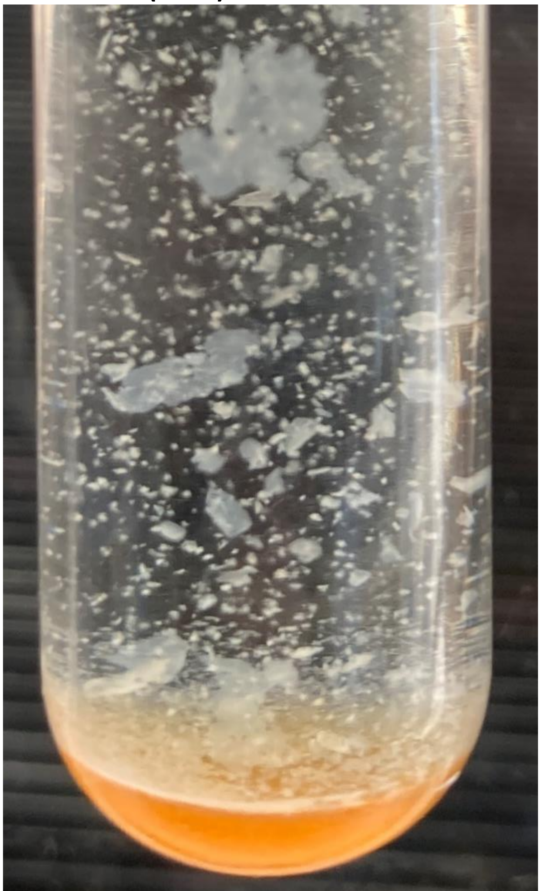

BCG-Danish (flake)

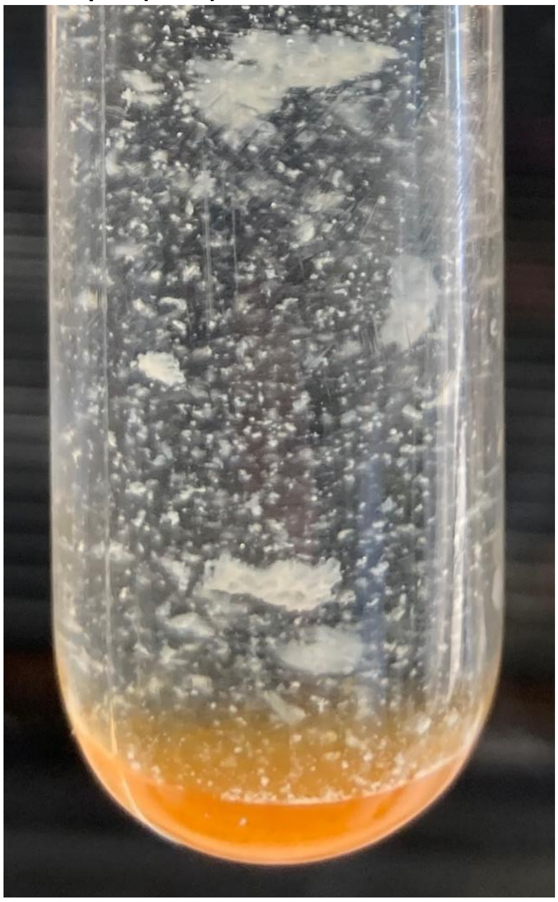

BCG-Prague (flake)

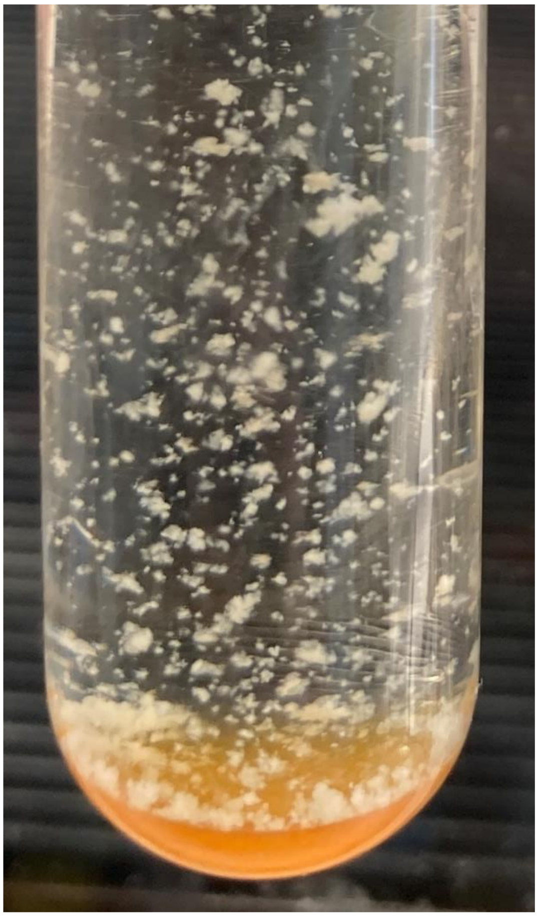

BCG-Glaxo (clump)

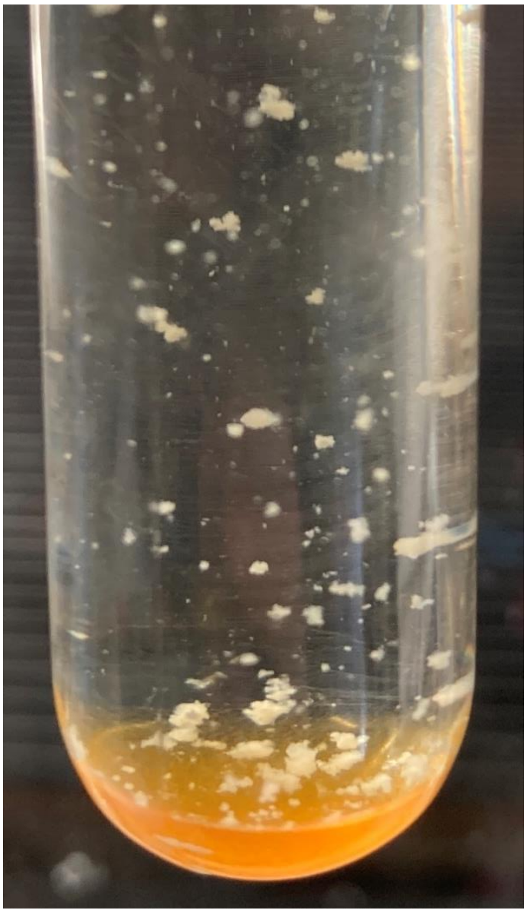

BCG-Pasteur (clump)

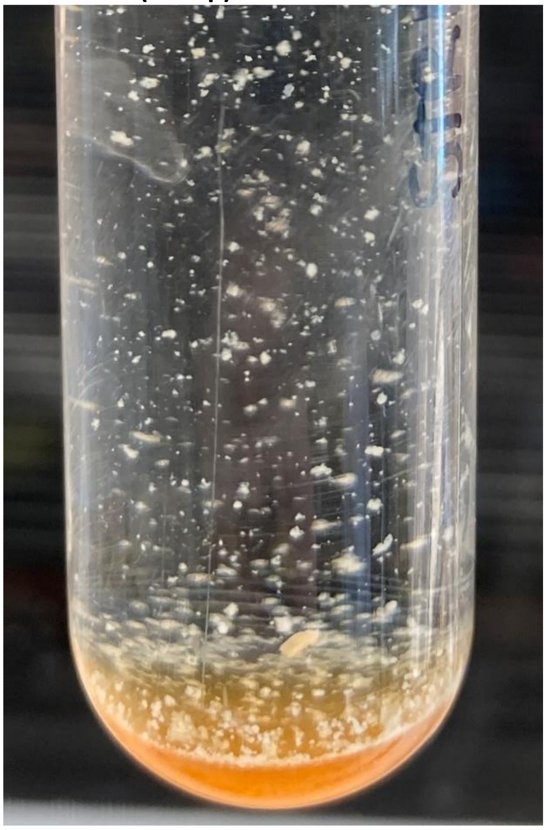

BCG-Dakar (clump)

Figure S3. Morphology of each geographically classified strain when grown in MGIT liquid culture.
